# A comparative genomic approach using mouse and fruit fly data to discover genes involved in testis function in hymenopterans with a focus on *Nasonia vitripennis*

**DOI:** 10.1186/s12862-021-01825-6

**Published:** 2021-05-19

**Authors:** Charlotte Lécureuil, Sophie Fouchécourt, Rémi Eliautout, Vanessa Guérin, Kevin Hidalgo, Dorian Neutre, Géraldine Roux, Philippe Monget

**Affiliations:** 1 Institut de Recherche sur la Biologie de l’Insecte (IRBI), UMR 7261, CNRS-Université de Tours, 37200 Tours, France; 2 PRC, CNRS, IFCE, INRAE, Université de Tours, 37380 Nouzilly, France; 3 INRA, UR633 Zoologie Forestière, 45075 Orléans, France; 4 COST, Université d’Orléans, Orléans, France

**Keywords:** Testis, Genes, Phylogeny, Hymenoptera

## Abstract

**Background:**

Spermatogenesis appears to be a relatively well-conserved process even among distantly related animal taxa such as invertebrates and vertebrates. Although Hymenopterans share many characteristics with other organisms, their complex haplodiploid reproduction system is still relatively unknown. However, they serve as a complementary insect model to *Drosophila* for studying functional male fertility. In this study, we used a comparative method combining taxonomic, phenotypic data and gene expression to identify candidate genes that could play a significant role in spermatogenesis in hymenopterans.

**Results:**

Of the 546 mouse genes predominantly or exclusively expressed in the mouse testes, 36% had at least one ortholog in the fruit fly. Of these genes, 68% had at least one ortholog in one of the six hymenopteran species we examined. Based on their gene expression profiles in fruit fly testes, 71 of these genes were hypothesized to play a marked role in testis function. Forty-three of these 71 genes had an ortholog in at least one of the six hymenopteran species examined, and their enriched GO terms were related to the G2/M transition or to cilium organization, assembly, or movement. Second, of the 379 genes putatively involved in male fertility in Drosophila, 224 had at least one ortholog in each of the six Hymenoptera species. Finally, we showed that 199 of these genes were expressed in early pupal testis in *Nasonia vitripennis*; 86 exhibited a high level of expression, and 54 displayed modulated expression during meiosis.

**Conclusions:**

In this study combining phylogenetic and experimental approaches, we highlighted genes that may have a major role in gametogenesis in hymenopterans; an essential prerequisite for further research on functional importance of these genes.

**Supplementary Information:**

The online version contains supplementary material available at 10.1186/s12862-021-01825-6.

## Background

The insect order Hymenoptera contains more than 150,000 described species and potentially around 850,000 undescribed species [1, 2]. This taxon includes all wasps, bees, and ants and represents approximately 8% of all described species [3]. Hymenopterans play a fundamental role in virtually all terrestrial ecosystems and make considerable economic contributions (e.g., in the form of biological control and pollination) [4]. Additionally, several wasp and bee species are of tremendous agricultural importance and are key components of global biodiversity: they provide vital ecosystem services to crops and wild plants and are involved in pest control [5, 6].

One of the most distinguishing characteristics of hymenopterans is haplodiploidy, a reproductive system in which diploid females arise from fertilized eggs and haploid males develop from unfertilized eggs [7]. Once a female has successfully mated, she stores the sperm in her spermatheca; consequently, one important factor constraining offspring production and sex ratio is the quantity and quality of sperm that females collect from males [8–10], which will determine the former’s ability to produce daughters in the future (even if female wasps can manipulate the sex ratio of their offspring to a certain degree [11–13]). The number of daughters produced is directly correlated with levels of parasitism by parasitoid wasps and with colony persistence in *Apis mellifera* because both phenomena rely solely on the activity of females. It is important to understand how haploid males produce sperm and to identify the genes involved in this process in order to study functional male fertility in hymenopterans, a group containing many species of agricultural significance.

**Fig. 1:**
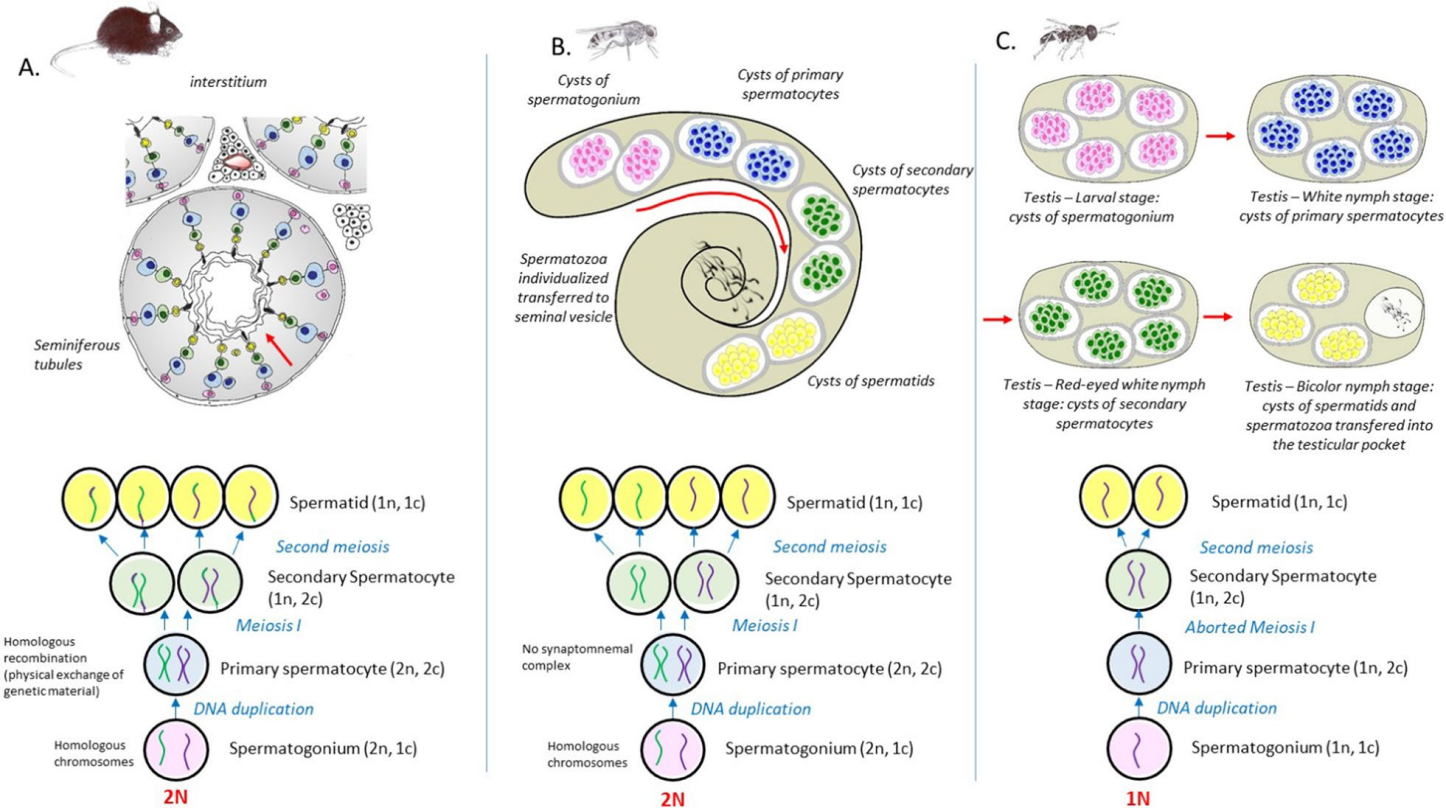
Schematic representation of testicular structure and spermatogenesis in adult mice (**A**), adult *Drosophila* (**B**), and in the various nymph stages of *Nasonia vitripennis* (**C**). Above: The spatial distribution of germ cells during these various stages is taxon specific: seminiferous tubule structure in mice (with the spermatogonia at the base of tubules and the spermatids in the luminal pole; see the red arrow) and cyst structure in the two insects. In *N. vitripennis*, each stage occurs consecutively during pupal development, whereas, in *Drosophila*, the stages occur simultaneously in the testis. Below: the main events occurring during meiosis, from the formation of spermatogonia to the formation of spermatids. The colors correspond to the four differentiation stages of spermatogenesis as follows: pink—spermatogonia (undifferentiated cells); blue—primary spermatocytes (after DNA duplication); green—secondary spermatocytes (after meiosis I); and yellow—spermatids (after meiosis II). In contrast to what occurs in the mouse, there is no recombination between homologous chromosomes in *Drosophila* (diploid) during meiosis. In *N. vitripennis,* males are haploid. Drawing of organisms © Eric Imbert/IRBI CNRS/Université de Tours

Spermatozoa are produced during spermatogenesis, which takes place in the testes. Spermatogenesis is a complex multi-phase process—the steps of mitosis, meiosis, and cytodifferentiation follow one another in an environment of shifting intercellular interactions. Spermatogenesis begins with a mitotic proliferation stage during which cytokinesis remains incomplete, leading to the creation of a cyst of interconnected germ cells. In the mammalian testis, germ cells are embedded in the epithelium in concentric layers; in contrast, in insects, germ cells form round spermatogenic cysts (Fig. 1). Meiosis is initiated by the production of primary spermatocytes that will, after DNA replication, undergo meiosis I (reduction division); during this process, homologous chromosomes pair up and the chromatids physically exchange genetic material. It is important to note that, in the fruit fly, there is no recombination, formation of the synaptonemal complex, or formation of chiasmata during spermatogenesis [14], which contrasts with what occurs in other diploid species. More importantly, in haploid male hymenopterans, this first meiotic phase is abortive [15, 16], and chromatid separation and recombination are impossible as males have a single set of chromosomes. In contrast, meiosis II (equational division) is more conventional in insects: the chromatids will be separated, and haploid spermatids will be produced. Then, spermiogenesis occurs, leading to chromatin remodelling and the formation of the flagellated tail.

Many genes are involved in the formation of mature spermatozoa. Based on microarray studies in mice (*Mus musculus*), more than 2,300 genes were found to be predominantly expressed in male meiotic and post-meiotic germ cells [17]. Moreover, several of these genes have been conserved through the course of evolution [18–20]. Across a large evolutionary scale, several genes have homologs that display similar testicular expression profiles [18]. In addition, experiments on model species such as mice, fruit flies, and worms have revealed the genes’ roles in regulating spermatogenesis [21].

**Fig. 2:**
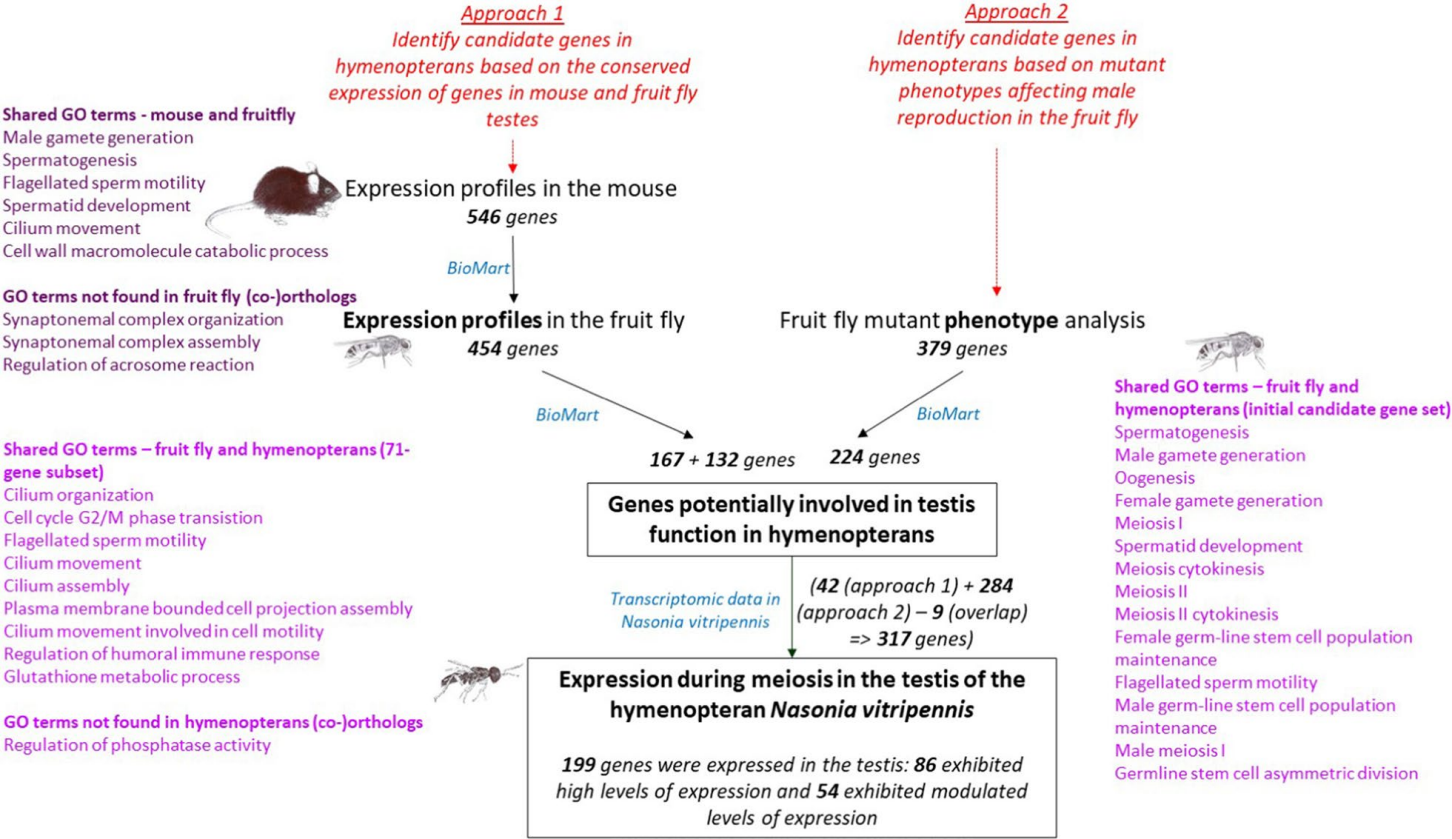
The two methodological approaches used to search for candidate genes involved in testis function in hymenopterans, with a focus on *Nasonia vitripennis*. GO terms for each gene list are provided (purple for the mouse and pink for the fruit fly). The numbers are in bold in the text. Drawing of organisms © Eric Imbert/IRBI CNRS/Université de Tours

**Fig. 3:**
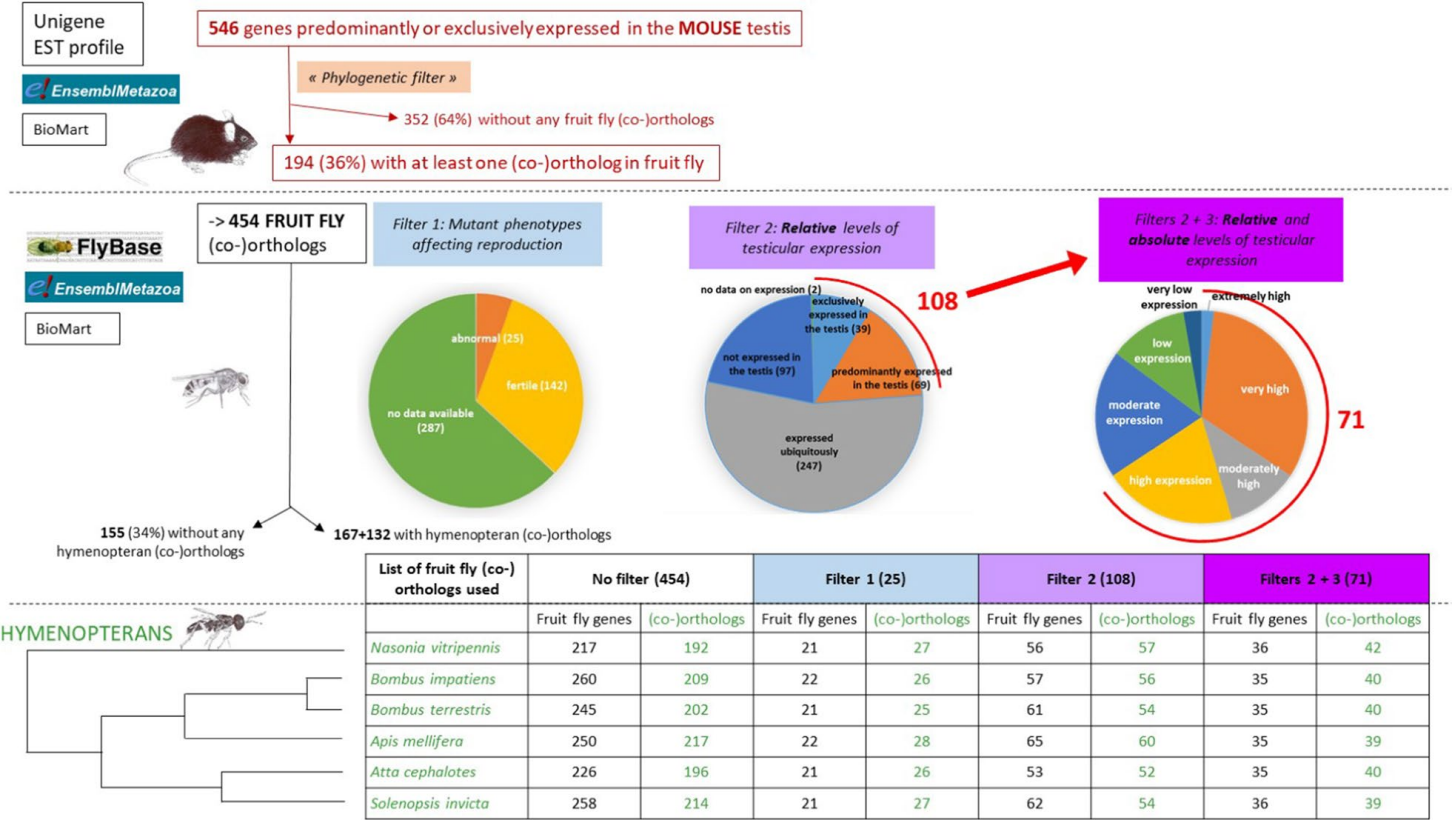
Schematic description of approach 1, which identified candidate genes based on high relative levels of testicular expression. Of the **546** mouse genes that were highly expressed in the testis, **454** fruit fly orthologs were found using the BioMart tool in Ensembl Metazoa (“Phylogenetic filter”). We applied three filters (filter 1: mutant phenotypes; filter 2: relative levels of testicular expression; filter 3: absolute levels of testicular expression for genes identified via filter 2) to the information in FlyBase to identify reproductive genes in *Drosophila*. Then, orthologs in hymenopterans were identified using the BioMart tool in Ensembl Metazoa and the various sets of fruit fly genes: the initial set of **454** genes and the subsets of **25** (filter 1), **108** (filter 2), and **71** genes (filters 2 + 3). The numbers are in bold in the text. The mouse data are in red, the fruit fly data are in black, and the hymenopteran data are in green. Drawing of organisms © Eric Imbert/IRBI CNRS/Université de Tours

In a previous study, we performed a phylogenetic meta-analysis of functional data from the fruit fly (*Drosophila melanogaster*) to identify genes with high expression levels in vertebrates (mice, zebrafish, and chicken), and we found that the expression of these genes was highly conserved in the testis (81%) [22]. Moreover, we showed there was a positive relationship between the genes’ functional relevance and levels of testicular expression. Here, our study is an approach without any a priori hypothesis concerning the gene functions that would be highlighted with such an approach, in which we aimed to use the abundant data available for model species to identify genes of importance for sperm production in hymenopterans. Candidate genes were identified based on information on gene expression and mutant phenotypes for mice and *Drosophila* that is found in major databases (i.e., Ensembl, Unigene, and FlyBase). We based our study on two approaches. First, we focused on genes that are predominantly expressed in the mouse testis, based on the hypothesis that high expression levels may reveal functional relevance; we subsequently searched for orthologs in the fruit fly (*D. melanogaster*) and in six hymenopterans (*Apis mellifera*, *Atta cephalotes*, *Bombus terrestris*, *Bombus impatiens*, *Nasonia vitripennis*, and *Solenopsis invicta*) (Figs. 2 and 3). Second, we used a list of fruit fly genes known to be involved in male reproduction to identify orthologs in the six hymenopterans (Fig. 2). We then used *N. vitripennis* as a representative hymenopteran species and evaluated the testicular expression profiles of some of the candidate genes.

## Results

### Approach 1: Identification of candidate genes in hymenopterans based on the conserved expression of genes in mouse and fruit fly testes

We identified **546** genes predominantly or exclusively expressed in the mouse testis using the EST profiles on the NCBI UniGene website (Additional file 1: Table S1), as described in the Materials & Methods. In the resulting list of mouse genes, 90% of the enriched GO biological process terms were associated with gametogenesis, which makes sense given the genes’ testis-specific expression (9 of the 10 GO terms were specifically related to reproduction; Additional file 1: Table S2). Among these **546** mouse genes, **194** (36%) had at least one ortholog in the fruit fly (Fig. 3), for a total of **454** fruit fly orthologs (Additional file 1: Table S3), based on the results of BioMart data mining. As a result, we found that 64% (352 genes; Fig. 3) of the genes exclusively expressed in the mouse testis did not have a fruit fly ortholog. Several of these genes had the enriched GO terms “synaptonemal complex organization or assembly” (4/11) and “regulation acrosome reaction” (4/16). Such genes (SYCE3, SYCE1, SYCP1, Hormad1, Spink2, Prss37, Fam170B, and IQCF1) may have appeared after the divergence of non-vertebrates and vertebrates or even after the appearance of mammals. However, other studies have found that a significant proportion of murine genes are absent from the genomes of model organisms like *Drosophila* or *Caenorhabditis* because they have been lost in these specific metazoan lineages [30].

### GO terms and testicular expression of fruit fly orthologs

**Fig. 4:**
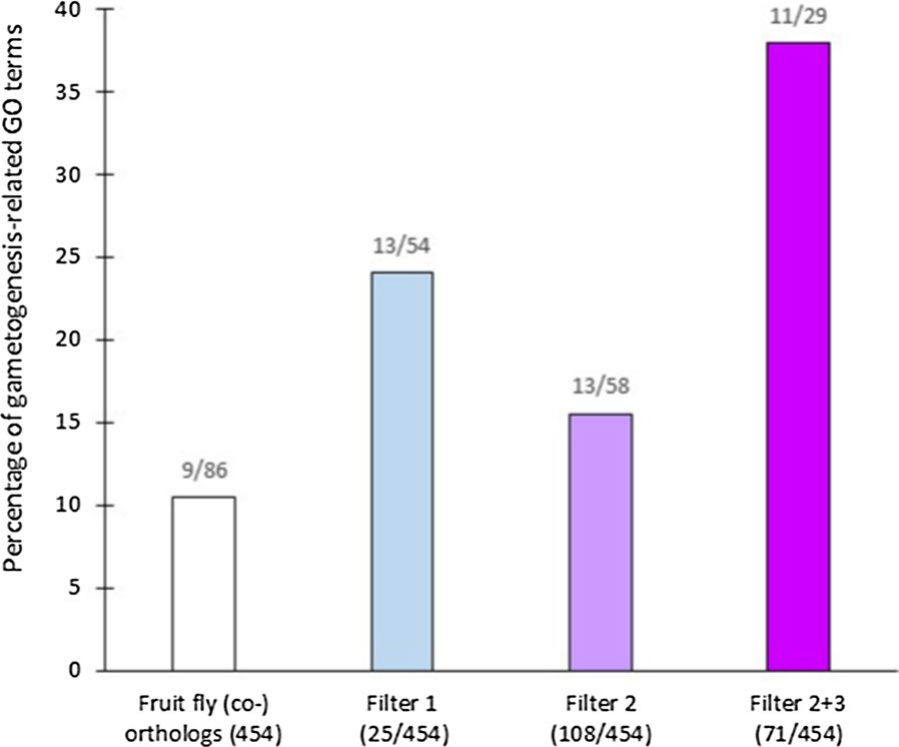
The percentage of gametogenesis-related GO terms among the GO terms with significant representation (p < 0.05) among the initial set of 454 *Drosophila* orthologs (gray) and the post-filtering subsets of genes; the absolute numbers are above the bars. Color coding: light purple—mutant phenotype filter (filter 1, **25** genes); intermediate purple—filter based on relative levels of testicular expression (filter 2, **108** genes); and dark purple—filter based on relative and absolute levels of testicular expression (filters 2 + 3, **71** genes). As we applied the successive filters, the GO terms were progressively enriched with terms related to gametogenesis

In the list of the **454** fruit fly genes, numerous enriched GO terms linked to metabolic pathways (glutathione/peptide/fatty acid metabolism) were present (Additional file 1: Table S4). Enriched GO terms related to gametogenesis (e.g., flagellated sperm motility, cilium organization) were also present (10%) but not predominant (Fig. 4). As orthologs can be linked to various non-reproductive functions, we targeted reproductive genes using three categories of FlyBase data as “filters”: data on (1) mutant phenotypes, (2) the relative levels of testicular expression (i.e., compared to other tissues), and (3) the absolute levels of testicular expression (see the pie chart in Fig. 3).

With regards to mutant phenotypes, male reproductive data were only available for 167 of the **454** genes. In the case of 142 of the genes, mutant fruit flies remained fertile (Additional file 1: Table S3: column H). However, in the case of the other 25 genes, mutations result in sterility (alphaTub84B, Ance, aub, blanks, bol, chic, ctp, CycB, CycD, fabp, hrg, klhl10, loqs, mael, mip120, mod, Ntl, PDZ-GEF, Pen, Ran, Sas-4, sfl, spn-E, TTLL3B, and tud). For these 25 genes, enriched GO Terms related to gametogenesis (e.g., spermatogenesis, oogenesis, female and male gamete generation) were predominant (24%) and were represented at a level that was 2.5 fold higher than the level seen in the whole list of **454** genes (13 of the 54 GO terms were specifically related to reproduction; Fig. 4 and Additional file 1: Table S5).

With regards to relative testicular expression, **108** of the **454** fruit fly orthologs exhibited expression that was either exclusive to (39) or predominant in (69) the testis compared to in other tissues (see the pie chart in Fig. 3 and Additional file 1: Table S3: column F). Mutations in 10 of these **108** genes cause sterility (aub, blancks, bol, CycB, Klhl10, loqs, mael, Ntl, Pen, and TTLL3B). GO term analysis revealed enrichment (16%; Fig. 4) in terms such as cilium organization/movement/assembly, flagellated sperm motility, and cell cycle G2/M transition (9 of the 58 GO terms were specifically related to reproduction, and they corresponded to 13 of the 108 genes; Additional file 1: Table S6). However, there are also various non-reproductive GO terms with very low P-values. To better pinpoint reproductive genes, we decided to focus on a subset of **71** genes of interest (15.5% of the initial set of **454** genes) that occurred among the **108** genes with marked relative levels of expression. However, these **71** genes stood out because their absolute levels of testicular expression were moderately high to very high (see the pie chart in Fig. 3 and Additional file 1: Table S3: column G). We hypothesized that this filtering choice would result in the selection of genes expressed in the germ cells that are preponderant in the testis (15 are testis specific and 56 are testis predominant). Indeed, these **71** genes had enriched GO terms (38%) related to cilia and flagella (11 of the 29 GO terms were specifically related to reproduction; Fig. 4 and Additional file 1: Table S7). Mutations in eight of these genes cause sterility (blanks, bol, CycB, klhl10, loqs, Ntl, Pen, and TTLL3B). Thus, thanks to the successive filters applied, the GO terms were progressively enriched with terms related to gametogenesis (Fig. 4).

### Hymenopteran orthologs and their expression in the testis and other tissues

Using the Ensembl Metazoa database, we searched for orthologs of the above **454** fruit fly genes in 6 hymenopteran species (*Nasonia vitripennis, Bombus impatiens*, *Bombus terrestris*, *Apis mellifera*, *Atta cephalotes*, and *Solenopsis invicta*) (Additional file 1: Table S3: columns I to N).

Among the **454** fruit fly genes, **155** genes (34%) had no orthologs in any of the hymenopteran species, **167** genes had orthologs in all 6 species, and **132** genes had orthologs in at least one of the six species (Figs. 2 and 3 as well as Additional file 1: Table S3: column O). For example, 217 fruit fly genes (48%) had at least one ortholog in *N. vitripennis*, resulting in a total of 192 orthologs.

Of the **71** fruit fly genes of interest, 26 genes (Additional file 1: Table S3: column O) had no orthologs in any of the hymenopteran species. The other 45 genes had at least one ortholog in at least one hymenopteran species, and their enriched GO terms were related to cilium organization, flagella, or sperm motility. Of these 45 genes, 28 had at least one ortholog in all six hymenopteran species. The other 17 genes had one or more orthologs in at least one of the hymenopteran species. The numbers of fruit fly genes and their orthologs in each of the six hymenopteran species are indicated in Fig. 3. When the focus was placed on *N. vitripennis*, there were 34 fruit fly genes (48%) with at least one ortholog, resulting in a total of **42** orthologs (Additional file 1: Tables S3: column I and S10).

### Approach 2: Identification of candidate genes in hymenopterans based on mutant phenotypes affecting male reproduction in the fruit fly

With the aim of finding additional genes involved in testis function in hymenopterans, we adopted a second approach. As a starting point, we used a list of **379** fruit fly genes known to be involved in male reproduction that we had previously established using FlyBase [22] (Fig. 2 and Additional file 1: Table S8). When we compared this list to the list of **454** genes from approach 1, there was only overlap in 23 genes (6%) (chic, ctp, CycB, klhl10, loqs, mip120, Ntl, Nup153, PDZ-GEF, Pen, piwi, Sas-4, sfl, spn-E, TTLL3B, tud, and mael). One thousand and ten enriched GO terms were found using this second list, and many reproduction-related genes were at the top of the list (Additional file 1: Table S9). Using the BioMart data mining tool, we found that, among these **379** genes, 75 had no orthologs in any of the 6 hymenopteran species, and **224** genes had at least one ortholog in each species (Additional file 1: Table S8: column J). When the focus was placed on *N. vitripennis*, there were 266 fruit fly genes with at least one ortholog, resulting in a total of **284** orthologs (Additional file 1: Tables S8: column D and S10).

Eleven genes known to be involved in meiosis in *N. vitripennis* appeared in our list of candidates: mei-P26 (NV10527), Incenp (NV22871), mip120 (NV11808), cdk1 (NV11863), cyclin B (NV11968), bol (NV13063), mip40 (NV13216), cyclin-A (NV14248), plk-4 (NV14781), plk-2/3 (NV15469), and taf6 (NV18184) [24, 27]. In the second approach, which utilized information on mutant phenotypes in the fruit fly, GO term enrichment analyses revealed 14 N*. vitripennis* genes involved in meiosis that had not been previously described: Rae (NV14833), dia (NV16014), pbl (NV11081), lam (NV11973), Sec8 (NV10567), twe (NV13132), sau (NV13806/NV19204), brun (NV15799), pelo (NV13121), Klp3A (NV11229), east (NV20170), Det (NV10358), Exo84 (NV18587/NV18385), and bol (NV13063). These genes are not necessarily exclusively expressed in the testis. It is for this reason that only cyclin B and bol were detected using both our approaches.

### Characterization of candidate gene expression in *Nasonia vitripennis*

To determine whether the *Drosophila melanogaster* genes displayed the same testicular expression profiles in *N. vitripennis*, we performed qRT-PCR to quantify the expression of five randomly selected genes. The latter were orthologs of fruit fly genes that were predominantly or exclusively expressed in the testis (i.e., part of the **71-**gene subset from approach 1) with an ortholog in each of the 6 species of hymenoptera and for which we could design efficient primers: lebercilin #NV11655, glutathione S-transferase O2 (GstO2) #NV12109, tubulin tyrosine ligase-like 3B (TTLL3B) #NV13119, tektin-1 #NV13124, and axonemal dynein light intermediate polypeptide (Adlip) #NV15130. We examined gene expression profiles in three body parts—the legs, the head, and the testis—at different stages of development representative of different germ cells. Four of the five genes (lebercilin, TTLL3B, tektin-1, and Adlip) displayed marked expression in the testis in *N. vitripennis*. One gene (GstO2) was expressed in the legs and head in addition to the testis (Additional file 2: Figure S1).

Finally, to clarify whether the **317** orthologs identified in *N. vitripennis* via the two approaches (approach 1: **42** genes + approach 2: **284** genes – **9** overlapping genes; Fig. 2) were expressed in the species’ testis, we assessed gene expression in a transcriptome of the testis from two early pupal developmental stages that were available in the lab: the white nymph stage and the red-eyed white nymph stage. During these 2 stages, the meiosis process takes place and spermatocytes II are observed in the cysts at the red-eye stage [23]. Although this analysis could not determine whether gene expression was testis specific or not, it could however reveal whether or not the gene was expressed in the testis. We found that **199** of the **317** genes were expressed in the testis during both stages, and **86** genes displayed high or very high absolute levels of testicular expression (Additional file 2: Table S10: column F). Of these **199** genes, **54** displayed modulated expression during meiosis: **22** were upregulated and **32** were downregulated during the first stage. Of the 22 upregulated genes, 19 caused infertility in the fruit fly when subject to induced mutations. Some of these genes were enriched in GO terms related to spermatid differentiation: armadillo (NV13691), didum (NV12941), cut up (13,256), and mule (16,259). However, most appeared to be involved in sperm motility: Dnai2 (NV10124), heat (NV10434), dynein (NV14122), Kl5 (NV16259), and dynein (NV16467). Levels of mRNA for these genes consistently increased slightly before the relevant processes began. Several of the 32 downregulated genes were enriched in the GO term “oogenesis”—Rab11 (NV10199), hop (NV16630), Nup154 (NV13560), Lis1 (NV12141), Neb (NV11667), Hsp83 (NV11936), pelo (NV13121), asp (NV11703), and Trl (NV15260)—as well as in the GO term “Notch receptor processing”—ruby (NV13459), carnation (NV16292), deep orange (NV17240), and garnet (NV11151). In contrast, 145 were expressed at similar levels during both stages. As for the 118 genes that were not expressed during early pupal development, it is possible that they are expressed during other developmental stages (larval, late pupal, or adult) that we did not explore in this study.

## Discussion

Past research in hymenopterans has identified genes involved in male reproduction. Some studies have examined the phylogenetic distribution of meiotic genes [24] and the sex-biased expression of genes [25]. Others have identified 156 protein-coding genes expressed solely in the testis [26, 27]. Using two original approaches, we identified additional candidate genes that are likely involved in male reproduction in hymenopterans. We based our first approach on comparisons of conserved gene expression profiles for the testes of the mouse and the fruit fly. In the second approach, we employed information on mutant phenotypes that affect male reproduction in the fruit fly. The limitations of the approach used in this study are mainly due to the availability of data at the genomic, assembly and annotation levels. On the other hand, the richest and most complete databases (mice and Drosophila used in this study) may be phylogenetically quite distant from the species of interest (here Hymenoptera) and may also lead to losses of information during the orthology search. Thus, the approach cannot be exclusive. Nevertheless, by using multiple filters (expression specificity, phenotype after mutation), it can be assumed that the genes found are few in number but well involved in testicular development in the species searched.

The idea guiding our first approach was that certain genes may display conserved testis-specific expression over evolutionary time as a consequence of selection pressure, highlighting the genes’ functional relevance. Our results show that, of the 454 fruit fly orthologs of genes predominantly or exclusively expressed in the mouse testis, 108 genes (24%) were also predominantly or exclusively expressed in the fruit fly testis. This result suggests that specificity in tissue expression can be well conserved between evolutionarily distant species (*Mus* and *Drosophila* diverged 643–850 MYA, according to an estimate from TimeTree [28]). It also indicates that there may be a conserved “signature of testicular expression" in the promoters of these genes. Within the class Insecta, we also showed that four (80%) of the five genes that were predominantly or exclusively expressed in the fruit fly testis had orthologs that were predominantly expressed in the testis of *N. vitripennis*. Nevertheless, these data should be treated with caution due to the low number of genes analysed and low number of biological replicates. MicroRNA (miRNA) may also be involved in the regulation of gene expression in the testis. Indeed, the analysis of the 454 fruit fly orthologs shows that mir-985 may have a regulatory role because it is associated with 92 out of the 2,584 genes in the DroID database (v. 2015). Mir-985 has already been classified as a male-biased miRNA transcript in the fruit fly [29].

We chose to use the fruit fly as the link between the mouse and the hymenopteran species for several reasons. First, currently available tools (the Ensembl toolkit and the Ensembl Metazoa database) cannot be used to directly explore the link between the two latter taxa. Second, a wealth of data is available on testicular gene expression patterns and functional phenotypes in the fruit fly, which can be useful for interpreting the functional results. Orthologs of mouse testis-specific genes can be linked to various non-reproductive functions. For example, the GO term “glutathione metabolic process” was highly enriched across the 454 fruit fly orthologs: 30 of the 47 genes in the total *Drosophila* genome associated with this term were found among these 454 genes. Although our gene list for the mouse contained just one gene, GSTO2 (Mm.63791), that represented this functional category, this single gene had 31 orthologs in the fruit fly. Based on gene expression profiles in the fruit fly, we determined that 28 of these orthologs were ubiquitously expressed or not expressed in the testis. There were three genes that were predominantly expressed in the testis: GstD9 (FBgn0038020), GstO1 (FBgn0035907), and GstE8 (FBgn0063492). The former two genes have at least one ortholog in hymenopterans. Because of the high evolutionary distance between the mouse and the hymenopterans, information about the levels and locations of gene expression in *Drosophila* made it possible to pinpoint the genes involved in reproduction.

Several pieces of information were used in this identification process. Enriched GO terms served as indicators. First, we selected genes that were known to affect male reproduction on the basis of mutant phenotypes (approach 2). While the result was an enrichment of the GO terms related to reproduction (associated with 11 genes), the list was drastically reduced in size, with only 25 genes remaining. This constraint was partly due to the absence of phenotypic information for 36% of the genes. Since the data on tissue expression were more complete, we applied a filter that was based on patterns of predominant or exclusive relative expression in the testis. This strategy excluded fewer genes and lead to the selection of 108 genes (24% of the initial list). Enriched GO terms related to reproduction were associated with 13 of the 108 genes. Additional filtering based on the absolute levels of testicular expression made it possible to further enrich the reproduction-related GO terms (associated with 12 genes) and focus on a subset of 71 genes. This GO-term-based approach was useful. It can be seen that, across all the lists, the GO terms only corresponded to a small proportion of the genes identified. Nevertheless, they were highly helpful as indicators of the term enrichment for a list, which must be considered in its entirety when selecting candidate genes. Using the first approach, we identified a total of 43 candidate genes that may be important in male reproduction in the six hymenopteran species included in our study.

Using our second approach, 379 genes were identified as potentially important in fruitfly because at least one of the mutants identified in flybase exhibited sterility or a reproductive defect in the male. This approach is not exclusive since the absence of a mutant in a database does not necessarily mean that the gene is not involved in male reproduction. The gene may be involved in vital functions before reproduction can be observed or it may not have been mutated in a functional area necessary for reproductive function. We found that 20% (75/383) of fruit fly genes do not have orthologs in any of the six hymenopteran species studied here. These genes were highly enriched in the GO terms “calcium-mediated signaling” (4/37) and “regulation of phosphatase activity” (2/12) but not in any terms related to reproductive processes. This result could suggest that these genes specifically appeared in the fruit fly or that they have been lost in hymenopterans. In any case, 22 of these genes have orthologs in the mollusk *Lottia gigantea*, and 18 have orthologs in the nematode *Caenorhabditis elegans.* The most recent common ancestor of dipterans and hymenopterans occurred about 340 MYA, and the most recent common ancestor of all hymenopteran species dates back to 190 MYA [31].

Only four of the genes were identified during a previous study that established a list of 153 testis-specific genes: TTLLB (NV13119), radial spoke head protein 6 homolog A (NV15436), Cyt-c-d (NV16243), and Ant2 (NV16915) [27]. However, they did not appear to be expressed during our transcriptome analysis. In contrast, we did observe the expression of genes that were on a germ cell-specific list: nudE (NV11388) and nos (NV18258) [27]. It is possible that such genes were not expressed during our transcriptome analyses because they are expressed during other developmental stages that were not explored here.

### Identification of highly conserved genes

By employing the two approaches in parallel, we identified nine genes in *N. vitripennis* (described below) that remain highly conserved between the mouse and the insect taxa—the fruit fly and the hymenopterans. These genes play a crucial role in male reproduction since their mutants displayed disrupted fertility and their expression levels were high in the testes of both the fruit fly and the mouse. Although these genes are known to occur in certain other species, this is the first time that they have been described in hymenopterans. Consequently, it would be useful to conduct complementary studies in this group, notably in *N. vitripennis*, a species for which we have only partial data on gene expression during spermatogenesis.

Among these nine genes, we found one gene whose expression was upregulated during meiosis: the kelch-like family member 10 (klhl10) (NV13442), which encodes a protein involved in spermatogenesis in *Drosophila* (FBgn0040038) [32, 33]. The protein recruits substrates for a testis-specific cullin-3-based E3 ubiquitin ligase complex (FBgn0261268), which is required for caspase activation during the terminal differentiation of spermatids (i.e., individualization) in the fruit fly. The cul-3 gene (NV11526) was not expressed in the testis of *N. vitripennis*. Moreover, cul-3’s partner in the fruit fly, Roc1b (FBgn0040291) [32], did not have an ortholog in any of the hymenopterans studied. As a result, it might be helpful to look for partners of klhl10 in *N. vitripennis*, genes that would be highly expressed in the testis and upregulated during development, as the species could have partners that are different from those in the fruit fly.

In contrast, two other genes displayed downregulated expression during meiosis in *N. vitripennis*: those encoding the double-stranded RNA-binding domain protein Blanks (NV18413) and Alpha 2 importin (NV14612), which are both proteins involved in sperm individualization. In the fruit fly, Blanks (FBgn0035608) is part of a nuclear siRNA/dsRNA-binding complex that plays a role in crucial RNA silencing-related pathways in the male germ line. It is only expressed in post-mitotic spermatocytes. Blanks expression continues as meiosis proceeds, and it ends with the onset of nuclear shaping. Loss of Blanks results in complete male sterility via a sperm individualization defect [34]. The alpha subunit of the importin belongs to a family of proteins that transports nuclear localization signal-bearing proteins inside the nucleus. Alpha 2 importin (FBgn0267727) is highly expressed and modulated during spermatogenesis. It is clearly involved in gametogenesis since induced mutations lead to fruit fly sterility—a large number of spermatids fail to individualize and remain syncytial [35].

Two of the nine genes were expressed in the testis, but their expression was not modulated during meiosis: the one encoding cyclin B (NV11968) was expressed at a very high level, and the one encoding Loquacious (NV14998) was expressed at a low level. Cyclin B is a well-known protein that binds to Cdk1, thereby promoting Cdk1 protein kinase activity. Cyclin B (FBgn0000405) is important in cell cycle transitions. During meiotic prophase in the male fruit fly, a transcript of Cyclin B1 is expressed in spermatocytes; however, the protein only begins to accumulate in the cytoplasm of spermatocytes just before the G2/MI transition [36]. Fest (FBgn0034435) is required to repress the translation of cyclin B in immature spermatocytes. The relevant gene (NV12142) was also found to be expressed in the testis of *N. vitripennis*, and its expression was not modulated during meiosis. Loquacious is a double-stranded RNA binding protein that associates with Dicer and thus facilitates the latter’s function in small RNA biogenesis. In the fruit fly, Loquacious is intrinsically required for germ stem cell renewal during oogenesis [37], but it is unknown if and how the loqs gene is involved in spermatogenesis.

The last four genes—those encoding boule (NV13063), neurotransmitter transporter-like Ntl (NV12838), tubulin tyrosine ligase like (NV13119), and Blanks (NV15099)—were not expressed in the testis of *N. vitripennis* during meiosis. Like cyclin B, boule (bol) is a well-known protein; it plays a crucial role in the transition to meiosis and in spermatid differentiation [38]. The bol gene was the first gene regulating critical steps of reproduction that was shown to be functionally conserved between the fruit fly (FBgn0011206) and humans [20]. In hymenopterans, which display incomplete meiosis I, a bol ortholog had already been found in *Apis* and *Nasonia* [24]. Furthermore, the functional importance of this gene has been shown in *Athalia rosae* (an ancestral hymenopteran) [39], a species in which it is highly expressed. Neurotransmitter transporter-like Ntl (FBgn0267326) is an important glycine transporter with testis-specific expression. Induced mutations in the gene encoding this protein result in male sterility in the fruit fly: mutants produce sperm with significantly reduced levels of glycylated tubulin and display an altered ability to transfer spermatozoa into the seminal vesicle [40]. This version of Blanks in *N. vitripennis* (NV15099) is the second known ortholog of Blanks in the fruit fly (FBgn0035608). TTLL3Bs are glycyclases that initiate the ATP-dependent addition of glycines to the internal glutamates of the tubulin tails in axonemes. In the fruit fly, low levels of TTLL3B (FBgn0031853) induce sterility by reducing axoneme stability in the final stages of spermatogenesis [41].

It is important to note that, in the fruit fly, there is no recombination, formation of the synaptonemal complex, or formation of chiasmata during spermatogenesis [14], which contrasts with what occurs in other species. In metazoans in general, the stable pairing of the homologous chromosomes is mediated by the assembly of the synaptonemal complex. Even if its tripartite structure is well conserved in metazoans, the proteins that comprise the synaptonemal complex are strikingly varied [42], and the complex found in ecdysozoans is markedly different from the complex found in other metazoans [43]. Consequently, genes involved in male recombination could not be identified in our study. Furthermore, one study showed that genes shared solely by honey bees and vertebrates (i.e., absent from dipterans) had expression profiles that were biased in the brain and testis [44]. Thus, it might be interesting in the future to analyze whether some genes involved in spermatogenesis are specific to hymenopterans. At present, the databases for hymenopterans are not comprehensive enough to use an approach similar to ours that does not employ *Drosophila* as an intermediate taxon.

## Conclusion

Using evolutionary distant organism models to explore spermatogenesis at the molecular level can help reveal the general underpinnings of this crucial process and highlight its conserved facets [45]. For example, adding hymenopterans to the pool of traditional model species could clarify how pesticides mechanistically affect non-target organisms. Using various biological data sources and several in silico and experimental tools, we found out genes that may have a major role in male reproduction in hymenopterans by focussing at 317 genes in *N. vitripennis*. Further research is now needed to verify the functional importance of these genes. Indeed, across animal species, more genes are expressed in testicular tissue than in any other tissue [46, 47]. One hypothesis for this pattern is that novel genes are more likely to appear in the testis because of the transcriptionally permissive chromatin state of germ cells [48], resulting in the predominant expression of these “new” genes in the testis or the male reproductive system. Some could be temporarily expressed in the testis and then evolve to acquire novel functions. Others, by contrast, could have a functional impact on spermatogenesis and may evolve under positive selection [49].

## Methods

As a general rule, the term “co-ortholog” is used when a given phylogenetic lineage has a gene for which there are two or more orthologs in another lineage. This may be the case for the genes in the present study, but for the sake of simplicity, we call orthologs all genes which have an orthology relationship, even if they are co-orthologs. We developed two phylogenetic approaches (Fig. 2) to identify hymenopteran genes related to male reproduction. In approach 1, the starting point was the creation of a list of mouse genes with testis-specific expression. We then searched for the orthologs of these genes in the fruit fly (Ensembl), which were then used in turn to identify orthologs in the six hymenopteran species (Ensembl Metazoa) (Fig. 3). In the second approach, the starting point was the list previously established by our lab [22] of 379 fruit fly genes for which there are mutant phenotypes displaying impaired spermatogenesis. We then examined the expression patterns of the final set of genes in hymenopterans (Fig. 4 and Additional file 1: Table S10).

### Identification of genes expressed in the mouse testis

As described in other studies [50, 50–53], we carried out in silico screening of genes predominantly or exclusively expressed in the mouse testis. Predominant expression in the testis meant that expression was stronger in testicular tissue than in other tissues. To perform the screening, we used the expressed sequence tag (EST) profile posted on the NCBI UniGene website. The site was retired in July 2019, but the data are still available on the FTP site (https://ftp.ncbi.nlm.nih.gov/repository/UniGene/). These data have been successfully used by other researchers to characterize male germ cell genes [54].

### Retrieval of insect orthologs

To retrieve the fruit fly orthologs or the hymenopteran orthologs (i.e., in Apis mellifera, Atta cephalotes, Bombus terrestris, Bombus impatiens, Nasonia vitripennis, and Solenopsis invicta) of the genes identified above, we used the BioMart data mining tool (http://metazoa.ensembl.org/biomart/martview/5153be1344faca3740ea9c457fc90744), which is part of the Ensembl toolkit and the Ensembl Metazoa database (last query: Ensembl Metazoa release 44); the methodology is described elsewhere [22]. The Enrichr database for mouse genes and the FlyEnrichr database for fruit fly genes (https://amp.pharm.mssm.edu/FlyEnrichr/) were used to determine gene ontology (GO) enrichment compared with the whole genome in mice or flies respectively [55, 56]. Briefly, the list of gene symbols was pasted in the “Input data” and submitted to “GO Biological Process 2018” in the “Ontologies” section. P-values are calculated via the standard statistical methods employed by most enrichment analysis tools: the Fisher's exact test or the hypergeometric test. It is a binomial proportion test that assumes that a binomial distribution exists and that the probability of any gene belonging to any given set is independent. FlyEnrichR uses the Benjamini-Hochberg (BH) procedure to account for this issue.

### Expression profiles of fruit fly genes

Using modENCODE transcriptome data for the fruit fly [57] from the FlyBase database (https://flybase.org/ release 6.27) [58], we obtained information about the expression of orthologous genes across different fruit fly tissues, including the testis. We then placed the genes in one of four categories based on their tissue expression profiles. These categories conveyed the relative levels of testicular expression for each gene:

(1) the “exclusively expressed in the testis” group contained genes expressed in the testis (regardless of expression level) and in the accessory gland and/or the imaginal disc and/or one other tissue at a level lower than “moderately high”; (2) the “predominantly expressed in the testis” group contained genes expressed in the testis at a level higher than the levels seen in any other tissues; (3) the “ubiquitously expressed” group contained genes expressed in the testis and in other tissues; and (4) the “not expressed in the testis” group contained genes that were not expressed in the testis. For genes in groups (1) and (2), we also qualitatively classified the absolute level of expression as extremely high, very high, high, moderately high, moderate, low, and very low, based on the scale used in FlyBase (i.e., in the “modENCODE Tissue Expression Data” tab). Finally, we identified male reproduction-related mutant phenotypes in the fruit fly using FlyBase (i.e., the “Phenotype” tab).

### Quantification of candidate gene expression in *Nasonia vitripennis*

Using a commercial kit (Nucleopin RNA XS, Macherey–Nagel, Duren Germany), total RNA was extracted from pooled samples of individuals (n = 60). We used heads and legs, as well as testicular tissue taken from individuals at four developmental stages (white nymph stage, red-eyed white nymph stage, bicolor nymph stage, and black nymph stage); these stages corresponded to different phases of spermatogenesis (Fig. 1), given that the latter process is synchronized in *N. vitripennis* [23]. Reverse transcription was performed with 500 ng of total RNA, which was quantified using a Qubit® 3.0 fluorometer (Thermo Fisher Scientific, Waltham, MA). An oligodT primer (Promega, USA) was employed with Omniscript Reverse transcriptase (Qiagen, Germany) in accordance with the manufacturer’s instructions. Samples of cDNA were obtained and stored at − 20 °C. PCR was carried out using a QuantStudio 6 Real-Time PCR system (Applied Biosystems, Foster City, USA) and a total PCR volume of 12.5 µL, with 3.125 µM of primers (Eurofins MWG Operons, Ebersberg Germany; Table 1). The specificity of the amplified fragments was verified by sequencing (amplicon sequencing services provided by Eurofins Genomics, Ebersberg, Germany), by evaluating amplicon size (Table 1), and by confirming that the melting curve contained a single peak. We verified that, for each set of primers, estimated efficiency was between 90 and 110%. To normalize the results, we used the genes rpl6 and rpl7 as internal standards and carried out relative quantitation using the Comparative CT method (delta-deltaCt) (User Bulletin #2 ABI PRISM 7700 Sequence Detection System, Applied Biosystems, updated 10/2001).

**Table 1:** Primer pairs used to characterize mRNA expression in *Nasonia vitripennis*

Locus	Identification *Nasonia* base	Abbreviation	Name	Forward primer	Reverse primer	Amplicon length
XM_001603114.2	NV15130	Adlip	Axonemal dynein light intermediate polypeptide 1	AGCAGATTTCAAGTACACCAGC	ATTCCAGTTTCTCGGGCTTG	100
XM_001605631.4	NV11655	lebercilin	Lebercilin-like protein	AGTCAGCTTTAGAAGGCGACCA	GTTATTGAGGTCGGCCTGTTGA	204
XM_001601569.3	NV13124	TEK1	Tektin-1	CATTAGCGCACACTAGATTGGG	CACAAGTGAGTTCCAAACCTGG	154
XM_008207383.2	NV13119	TG3A	tubulin glycylase 3A-like	ATGGTCTTTCAGAGAGGCAC	ATTAAGTCGCTCGCAATCCC	154
NM_001172445.1	NV12109	GSTO2	Glutathione S-transferase O2	TTGTTCTGAGCAACCACCAC	AGCTGGAACCTTGCCTTCTG	187
NM_001159919	NV12167	Rpl6	Ribosomal protein L6	AAGAAGACACCCAAGAAGGAA	ACAATGGGATCTGAGGTAGGA	137
NM_001159854	NV17492	Rpl7a	Ribosomal protein L7a	AAGAAAGTCGAGCCCAAGAAG	GGCTGAATATCCTCGGCAAT	80

### Transcriptome analysis

#### Testis dissection and total RNA extraction

The transcriptome analysis was performed using the testes of *N. vitripennis* pupae at two developmental stages: the white nymph stage (during which the primary spermatocytes are the only germ cells present) and at the red-eyed white nymph stage (during which the secondary spermatocytes are the only germ cells present) [23]. To represent each stage, the testes of 50–60 males (i.e., 100–120 testes) were dissected in RNA-free water, immediately snap frozen in liquid nitrogen, and stored at -80 °C until RNA extraction could take place. Three pools per condition were used. RNA was extracted from the samples using a NucleoSpin® RNA XS Kit (Macherey–Nagel gmbH & Co. KG, Germany) and an adapted version of the manufacturer’s instructions. The RNA extractions were then eluted in 11 µl of RNAse-free water and stored at -80 °C until the transcriptome analysis took place. Total RNA concentration was quantified using a Qubit® 3.0 fluorometer (Thermo Fisher Scientific, Waltham, MA) and a NanoDrop spectrophotometer (Thermo Fisher Scientific, Waltham, MA, USA). RNA integrity was verified using an Agilent 2100 Bioanalyzer (Agilent, Santa Clara, CA, USA).

Samples were sent to GenoScreen (Lille, France) for sequencing. Sample quality was verified beforehand using a Quant-iT™ RiboGreen® RNA Assay Kit (Thermo Fisher Scientific, Waltham, MA, USA), a DNF-471 RNA Analysis Kit (15 nt, standard sensitivity; Advanced Analytical Technologies, Ankeny, USA), and a fragment analyzer.

To generate RNA-seq libraries, the Illumina TruSeq RNA Sample Preparation Kit (Illumina, San Diego, California) was used in accordance with the manufacturer’s instructions. Briefly, mRNA was purified from 200 ng of the total RNA from each sample, enriched using polyT marble, and fragmented. Then, mRNA was converted into double-stranded cDNA. Sequencing barcodes were ligated to the cDNA fragments, and the resulting fragments were amplified using PCR. Libraries were validated using the DNF-474 High-Sensitivity NGS Fragment Kit and a fragment analyzer to confirm that the libraries had the expected fragment size of ~ 350 bp. Finally, SYBR™ Green I dye was used to ensure adequate library concentrations.

Sequencing libraries were multiplexed such that each multiplex contained one library from each of the two developmental stages and were then sequenced using a HiSeq 2500 System (rapid run mode, 2 × 100 bp; Illumina, San Diego, California). For each sample, we obtained sequences ranging from 1,040 to 3,136 Mbp.

#### Mapping and counting reads

Reads were mapped to the annotated reference genome of *N. vitripennis* [59] (Nvit_2.1; obtained from [www.ncbi.nlm.nih.gov/] using Tophat2 [60], which is a short-read aligner that can identify and predict splice junctions between exons and that is available on the Galaxy platform (https://usegalaxy.org/). Alignment files were then processed by sorting the SAM files. Counts were carried out using the htseq-count script that is part of the HTSeq python module, which determines the total number of sequencing reads that are aligned with each putative gene model in the *N. vitripennis* reference genome (Additional file 1: Table S11). To determine which testis genes were differentially expressed across the different developmental stages, we used SARTools, a DESeq2 R pipeline available on the Galaxy platform [61]. We identified the genes that were differentially expressed between the two developmental stages using the fold-change values and the Benjamini–Hochberg method of post-hoc comparisons (adjusted p-value < 0.05).

## Supplementary Information

**Additional file 1: Table S1.** List of the **546** mouse genes expressed exclusively or predominantly in testes according to the Unigene “EST profile” database used for the approach 1, and their (co)-orthologs in Drosophila melanogaster retrieved with the Biomart tool. **Table S2.** Enriched « Biological Process» GO Terms for mouse genes expressed exclusively or predominantly in the testis (n = 447) (adjusted p-value < 0.05). **Table S3.** List of the **454** co-orthologs in Drosophila melanogaster, with their relative expression in testis (compared to other tissues), their absolute level of expression in testis and their phenotype when mutated according to Flybase database. Their orthologs in six hymenopterans (EnsemblMetazoa Biomart tool) are listed in columns I to N, with a summary in column O. **Table S4.** Enriched « Biological Process» GO Terms for Fruit fly orthologs of mouse genes expressed exclusively or predominantly in the testis (adjusted p-value < 0.05). **Table S5.** Enriched « Biological Process» GO Terms for the **25** Fruit fly orthologs with a abnormal reproductive phenotype after mutation (filter 1) (adjusted p-value < 0.05). **Table S6.** Enriched « Biological Process» GO Terms for the **108** Fruit fly orthologs with a related expression in testis predominat or specific (filter 2) (adjusted p-value < 0.05). **Table S7.** Enriched « Biological Process» GO Terms for the **71** Fruit fly orthologs with a specific or predominant relative expression in testis and an absolute level of expression in testis at least moderatly high (filters 2 + 3) (adjusted p-value < 0.05). **Table S8.** List of the **379** Fruit fly genes involved in male reproduction according to their mutant phenotype according to Flybase and and retrieved in our previous work (Fouchécourt et al., 2019), and their co-orthologs in the six hymenoptera according to the Biomart tool. **Table S9.** Enriched « Biological Process» GO Terms for Fruit fly genes involved in male reproduction according to their mutant phenotype according to Flybase. **Table S10.** List of genes potentially important for male reproduction in *Nasonia vitripennis* identified with both approaches, and their expression in the nymphal testis determined by a transcriptomic analysis between testes of white nymphae and white nymphae with red eyes. **Table S11.** Transcriptomic informations on data used for the analysis.

**Additional file 2: Figure S1.** Relative mRNA expression levels of the 5 candidate genes in the testis (developmental stages: white stage, white stage with red eyes, bicolor stage, and adult), legs, and head of *Nasonia vitripennis* (n = 60 individuals) as determined by qRT-PCR (reference = levels of testicular expression during the white nymph stage). These results are representative of the numerous other experiments carried out with these biological samples.

## Data Availability

All data generated and analyzed during this study are included in this published article as figures or in the supplemental files including figures and tables. The data discussed in this publication have been deposited in NCBI's Gene Expression Omnibus (Edgar et al., 2002) and are accessible through GEO Series accession number GSE160291 (https://www.ncbi.nlm.nih.gov/geo/query/acc.cgi?acc=GSE160291).

## Abbreviations

DNA: Deoxyribonucleic acid
EST: Expressed sequence tag
FTP: File transfer protocol
GO: Gene ontology
KEGG: Kyoto Encyclopedia of Genes and Genomes
klhl10: Kelch-like family member 10
Mbp: Megabase pairs
MYA: Millions of years ago
miRNA: Micro ribonucleic acid
NCBI: National Center for Biotechnology Information
Ntl: Neurotransmitter transporter-like
oligodT: Oligodeoxythymidylic acid
PCR: Polymerase chain reaction
RNA: Ribonucleic acid
rpl6: Ribosomal protein L6
