# Supplementary material for: A comparative genomic approach using mouse and fruit fly data to discover genes involved in testis function in hymenopterans with a focus on Nasonia vitripennis
Source: BMC Ecol Evol. 2021 May 19;21:90. doi: 10.1186/s12862-021-01825-6 (PMC8132408; doi:10.1186/s12862-021-01825-6)
Supplement: Supplementary file 2 — Additional file 2: Figure S1. Relative mRNA expression levels of the 5 candidate genes in the testis (developmental stages: white stage, white stage with red eyes, bicolor stage, and adult), legs, and head of Nasonia vitripennis (n = 60 individuals) as determined by qRT-PCR (reference = levels of testicular expression during the white nymph stage). These results are representative of the numerous other experiments carried out with these biological samples. [file 12862_2021_1825_MOESM2_ESM.docx]

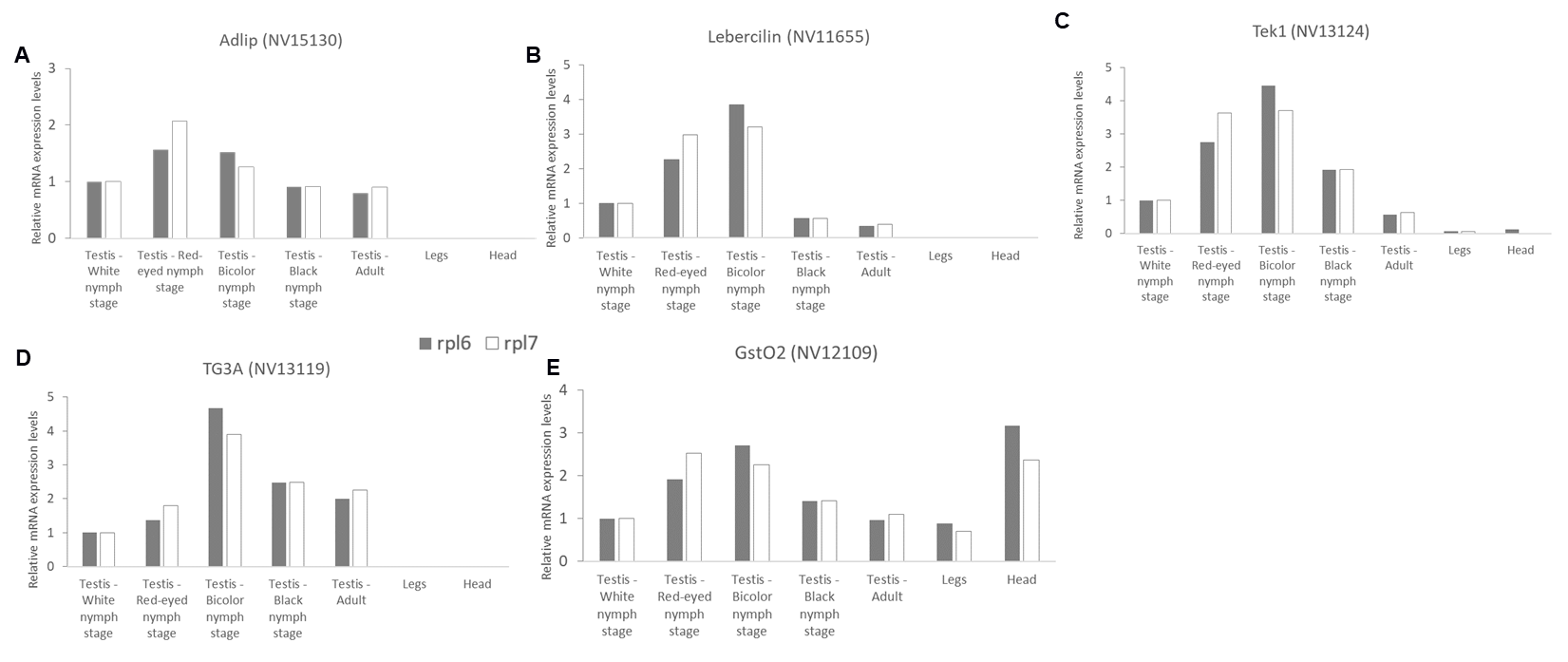


**Figure S1:** Relative mRNA expression levels of the 5 candidate genes in the testis (developmental stages: white stage, white stage with red eyes, bicolor stage, and adult), legs, and head of *Nasonia vitripennis* (n = 60 individuals) as determined by qRT-PCR (reference = levels of testicular expression during the white nymph stage). These results are representative of the numerous other experiments carried out with these biological samples
